# Metagenomic Analysis Reveals Presence of *Treponema denticola* in a Tissue Biopsy of the Iceman

**DOI:** 10.1371/journal.pone.0099994

**Published:** 2014-06-18

**Authors:** Frank Maixner, Anton Thomma, Giovanna Cipollini, Stefanie Widder, Thomas Rattei, Albert Zink

**Affiliations:** 1 Institute for Mummies and the Iceman, EURAC Research, Bolzano, Italy; 2 CUBE - Division of Computational Systems Biology, Department of Microbiology and Ecosystem Science, University of Vienna, Vienna, Austria; Argonne National Laboratory, United States of America

## Abstract

Ancient hominoid genome studies can be regarded by definition as metagenomic analyses since they represent a mixture of both hominoid and microbial sequences in an environment. Here, we report the molecular detection of the oral spirochete *Treponema denticola* in ancient human tissue biopsies of the Iceman, a 5,300-year-old Copper Age natural ice mummy. Initially, the metagenomic data of the Iceman’s genomic survey was screened for bacterial ribosomal RNA (rRNA) specific reads. Through ranking the reads by abundance a relatively high number of rRNA reads most similar to *T. denticola* was detected. Mapping of the metagenome sequences against the *T. denticola* genome revealed additional reads most similar to this opportunistic pathogen. The DNA damage pattern of specifically mapped reads suggests an ancient origin of these sequences. The haematogenous spread of bacteria of the oral microbiome often reported in the recent literature could already explain the presence of metagenomic reads specific for *T. denticola* in the Iceman’s bone biopsy. We extended, however, our survey to an Iceman gingival tissue sample and a mouth swab sample and could thereby detect *T. denticola* and *Porphyrimonas gingivalis*, another important member of the human commensal oral microflora. Taken together, this study clearly underlines the opportunity to detect disease-associated microorganisms when applying metagenomics- enabled approaches on datasets of ancient human remains.

## Introduction

Next generation sequencing (NGS) provides a unique opportunity to address fundamental research questions in various scientific fields [1]–[3]. Numerous studies have applied NGS to reconstruct the genomes of different lifeforms [4]–[6] and have used this high-throughput methodology to analyse the genetic anomalies in human cancers [7] or to resolve the structure and function of the human microbiome [8], to name two examples.

In addition to this, the field of ancient DNA research has also benefitted from advancements in sequencing technology. Highly degraded ancient DNA [9], [10], with a majority of fragments having a size of 50 bp or smaller, cannot be fully targeted by conventional polymerase chain reaction (PCR) and/or Sanger-based sequencing. However, NGS technologies that produce read lengths between 35 and 400 bp [11] cover this small size sequence range. Thus, highly fragmented ancient DNA, present in minute amounts, can be analyzed to an unprecedented depth and accuracy with NGS. Draft nuclear genomes of a 38,000-year-old Neanderthal specimen [12] and of the 30,000 to 48,000-year-old Denisova fossil [13] display milestones in the application of NGS on ancient remains and shed new light into the genetic evolution of hominoids. Further NGS based studies have allowed new insights into the phenotype and origin of the 4,000-year-old Saqqaq individual [14] and the mummified 5,300-year-old Iceman [15]. In the near future NGS will be routinely used to analyze ancient human remains. The more ancient human genomes become available, the more we will then learn about our population history [16]. The aforementioned ancient hominoid genome studies can be regarded by definition as metagenomic analyses since they represent a community of both hominoid and microbial sequences in an environment. The amount of non-hominoid sequence material can thereby vary dramatically ranging from 15.8% of all recovered reads in the Shaggaq genome study [14] up to 99% of all reads in the Neanderthal genome study [12]. This percentage of recovered reads in metagenomes is not only affected by the complexity and diversity of the community but also by the sequencing depth.

Initially, these non-hominoid sequence reads had been declared as complex background dataset and were not subjected to further in-depth analysis. Several recently published studies, however, impressively demonstrate what precious additional information besides the human host genome can be extracted from ancient DNA datasets of human remains. The combination of DNA enrichment methodology with NGS enabled researchers to recover the first complete ancient pathogen genome of *Yersinia pestis* from Black Death victims in Medieval England [17]. In one exceptionally well-preserved medieval individual with indicative leprosy bone lesions, it was even possible to *de novo* assemble the complete genome of an ancient *Mycobacterium leprae* strain from the ancient DNA dataset without previous DNA enrichment [18]. Both studies provided new insights into the evolution of important human diseases as these applied methodologies paved new ways for analyzing ancient DNA datasets.

Despite the reconstruction of ancient pathogen genomes in individuals with known pathologies, the analysis of ancient DNA datasets also offers the potential to detect unexpected, ancient pathogens without any previous pathological indication. The analysis of the non-human reads in the Iceman’s genome study, for example, indicated the presence of *Borrelia burgdorferi* sequence reads [15]. Recently, the re-analysis of the Iceman’s metagenomic dataset using a different bioinformatics pipeline proved the presence of *Borrelia* reads on the genus level [19]. Further SNP based analysis, however, indicated a *Borrelia* species divergent form *B. burgdorferi*. This different species assignment displayed how challenging it can be to identify and correctly classify endogenous ancient DNA in huge datasets of highly fragmented DNA reads and it furthermore stresses the necessity for accurate filtering steps and computational proof for ancient DNA authenticity [20], [21]. For the taxonomic classification of metagenomic reads, the most basic method is to use BLAST to search for homology-based similarity to references in a database. Most previous studies provided a taxonomic profile of the non-hominoid sequence reads by using a BLASTn search against the public database [15], [22]. This basic approach, however, is known to be error prone and can lead to false positive assignments [19]. The study of Zaremba-Niedz’wiedzka and Anderson [23] provided an alternative approach in taxonomically profiling the Neanderthal metagenome by BLAST searching against a designated rRNA gene sequence database. The specific sequence curation of rRNA databases and the high phylogenetic resolution of the rRNA genes allow a relatively fast read alignment and accurate taxon assignment. In addition, Zaremba-Niedz’wiedzka and Anderson’s [23] computationally age classified the retrieved rRNA reads which finally brought the authors to the conclusion that the microbiome of the Neanderthal bone sample was highly dominated by *Actinobacteria* which showed no sign of DNA damage.

In this study we re-analyzed the metagenomic data of the Iceman’s genomic survey. Similar to the study of Zaremba-Niedz’wiedzka and Anderson [23] we first taxonomically profiled the non-human dataset by screening for rRNA specific reads. Unexpectedly, the taxonomic assignment and further genome wide analysis indicated the presence of sequence reads most similar to a human opportunistic oral pathogen. The sequence reads were then further subjected to DNA damage analysis and used for phylogenetic assignment. Finally, we extended our survey to detect members of the human commensal oral microflora in the Iceman’s oral cavity.

## Materials and Methods

### *In silico* Analysis of the Iceman’s Metagenome

#### Identification of the non-human reads in the iceman’s metagenome

All SOLiD sequence reads from the Iceman’s whole genome sequencing study ([15]; ENA Study Accession No.: ERP001144) were filtered for a minimal mean quality score of 20 and, in an additional step, for paired reads both passing the mean quality filter. To separate human from non-human reads, all remaining read pairs were mapped against the human reference genome hg19 GRCh37 (Feb. 2009, GB Accession No.: GCA_000001405.1) using the SHRiMP software package [24] with the following parameters: -p opp-in -I 10,1000-n 3-I −6, reflecting an insert size of 10 to 1000 nucleotides for the paired-end reads and a mismatch score of −6 (see Appendix S1 for details on the parameter optimization). In this manner, the retrieved unmapped reads represent the non-human fraction of the Iceman metagenome. In all further analysis, we refer to this dataset as “Iceman non-human reads”.

#### Taxonomic profile of the iceman’s non-human reads and screening for potential pathogens

The taxonomic structure of the metagenome was determined from all Iceman non-human reads that encode segments of the small subunit (SSU) or large subunit (LSU) ribosomal RNA (rRNA) genes, using sequence alignment and taxonomy assignment parameters optimized for the degraded DNA present in the Iceman sample. The Iceman non-human reads were searched against the SILVA database (SSU/LSU ref SILVA 106 from 30th august 2011; [25]). The BLAST [26] search was performed with the following parameters: -b 1000-v 1000-e 0.1-F F. The BLAST hits were further analysed with the MEGAN 4 software package [27] and assigned according to their taxonomy based on the SILVA database. The profile was created with the options: synonyms file = silva2ncbi.map (from http://www-ab.informatik.uni-tuebingen.de/data/software/megan4/download/silva2ncbi.zip); usekegg = false; useseed = false. The scoring parameters were: maxmatches = 1000 minscore = 50 toppercent = 10 minsupport = 1. The abundance of bacterial genera was obtained from the resulting taxonomic profile of the Iceman non-human reads. Each genus present in the metagenome was classified according to the NCBI microbial genome database (ftp://ftp.ncbi.nih.gov/genomes/genomeprj/lproks_0.txt). Genera containing mainly human-pathogenic bacteria represent reasonable candidates for Iceman-associated pathogens and were therefore selected for further analysis at the species level.

#### Determination of reads originating from treponema genomes

All non-human Iceman metagenomic reads were mapped against all available complete genomes of the genus *Treponema* in the NCBI RefSeq database [28]. This mapping was performed with SHRiMP using the following parameters for unpaired mapping, reduced mismatch score for more sensitive mapping of reads, and reporting only the best mapping alignment: -n 2-i 6–report 1. To prevent unspecific alignments resulting from close sequence similarities between *Treponema* and non-*Treponema* genomes, we retained only reads having sufficiently better similarities to *Treponema* compared to non-*Treponema* genomes (details in Appendix S1). Finally, all *Treponema* specific reads were extracted from the mapping file and used for further analysis.

#### Reconstruction of ancient genomic fragments of treponema denticola

For the *Treponema denticola* ATCC 35405 genome (NCBI GenBank accession AE017226.1), to which most of the *Treponema*-specific reads were most similar, contiguous consensus sequences were extracted using the mpileup command implemented in samtools [29]. For each genomic region, the gene coordinates and names were obtained from the genome annotation of the *T. denticola* ATCC 35405 genome. For further phylogenetic assignment, the contig containing the 23S rRNA gene was extracted. The logarithmic coverage of the genome was plotted against CDS regions (up- and downstream) and against the tRNA and rRNA regions. The circular plot was done using DNAPlotter from the Artemis package [30].

#### mapDamage analysis

To assess the nucleotide misincorporation patterns along the DNA fragments, we performed a mapDamage analysis [20] using all reads mapped to the *T. denticola* ATCC 35405 reference genome (gi|41821838|gb|AE017226.1|). Results were compared to the mapDamage patterns of the Iceman genome specific reads and reads of the human reference genome HG00101 (ENA Study Accession No.: SRP001294), which was also sequenced on a SOLID platform. The map step integrated in the mapDamage tool was performed using the following parameters: −l 70-a 10-t 4. In the plot step using the parameters −l 50-m 0.1, the y-axis range displaying the misincorporation frequencies was set to 0.5 for *Treponema* and 0.1 for the Iceman and human reference samples to display the full range of nucleotide misincorporation frequencies in the different datasets.

### Phylogenetic Assignment of the *Treponema denticola* 23S rRNA Contig

The sequence analysis and phylogenetic assignment of the 23S rRNA containing contig was performed with software tools implemented in the ARB software package [31]. The partial Iceman *Treponema* 23S rRNA contig was aligned against a subset of complete 23S rRNA sequences of the genus *Treponema* within the aligned SILVA large subunit ribosomal RNA dataset (SILVA 111, LSU Ref) [32]. The alignment was afterwards manually refined using the ARB sequence editor. Phylogenetic analyses were performed using the DNA maximum-likelihood method [PhyML [33] with the JTT substitution model] implemented in the ARB software package. The partial Iceman *Treponema* 23S rRNA contig was added to the phylogenetic tree using the ARB Parsimony tool with a filter on the 107 informative positions of the contig. Selected non-*Treponema* sequences of the phylum *Spirochaetes* served as outgroups.

### Iceman’s Gingival Tissue and Mouth Swab Samples

To further screen for opportunistic oral pathogens using PCR, a gingival tissue biopsy (1321) and a mouth swab sample (1324) were taken from the right inner Iceman’s mouth region (Fig. S2). Samples were withdrawn using a bone tissue biopsy needle (TRAPSYSTEM®SET, TRAPJ0810, HS Hospital Service S.P.A., Rome, Italy) for sample 1321 and by using a swab sampling device (Copan Diagnostics Inc., California, USA) for sample 1324. The sampling took place under sterile conditions at a temperature of 4°C in the Iceman’s conservation cell at the Archaeological Museum of Bolzano, Italy. The samples were immediately stored at −20°C in the ancient DNA laboratory of the EURAC - Institute for Mummies and the Iceman.

### Molecular Screening for Opportunistic Oral Pathogens

The Iceman’s gingival tissue and mouth swab samples were further subjected to molecular paleomicrobiological analysis to screen for the opportunistic oral pathogens *T. denticola* and *Porphyrimonas gingivalis.* The molecular analyses were conducted at the ancient DNA Laboratory of the EURAC - Institute for Mummies and the Iceman, Bolzano, Italy. Sample preparation and DNA extraction was performed in a dedicated pre-PCR area following the strict procedures required for studies of ancient DNA: use of protective clothing, UV-light exposure of the equipment and bleach sterilization of surfaces, use of PCR workstations and filtered pipette tips. DNA extraction was performed with approximately 40 mg of gingival tissue and 300 µl swab sample using a chloroform-based DNA extraction according to Tang and colleagues [34] with minor modifications. Three sets of newly designed primers were used in the PCR assay (Table S1). For the 16S rRNA gene PCR assay we adapted an already existing PCR based detection method for *T. denticola* [35] (for details please refer to Table S1) by shortening the fragment length from 316 bp to 68 bp. We took the forward primer from the original publication [35] and manually designed a new reverse primer in a *T. denticola* specific region by using the ARB editor of the ARB software package and the SILVA small subunit ribosomal RNA dataset (SSURef_106_SILVA). The two newly designed IS1126 primer pairs target the repetitive element IS1126 of *P. gingivalis* by amplifying a fragment of 71 bp and 98 bp length respectively. The PCR reaction mix for all primer sets contained 10 mM Tris-HCl (pH 8.3), 50 mM KCl, 1.875 mM MgCl_2_, 200 µM of each deoxynucleotidetrisphosphate, 0.5 µM of each primer, 0.1 mg/ml bovine serum albumin, 0.05 U/µl AmpliTaq Gold (Applied Biosystems, Foster City, CA, USA) and 4 µl of extracted DNA to a final volume of 50 µl. Polymerase chain reaction was carried out according to the parameters in Table S1. The PCR products were initially documented by electrophoresis on 2.8% agarose TBE gels and then either used directly for Sanger sequencing or cloned into the pCR 2.1-TOPO vector (Life Technologies, Carlsbad, CA, USA) prior to sequencing. Subsequently, 5 µl of direct PCR product or M13-based PCR amplification product from the vector were treated with 1 U of Shrimp Alkaline Phosphatase (SAP) and 0.8 U of ExoI and incubated at 37°C for 60 min, followed by heat inactivation at 75°C for 15 min. Four microliters of each reaction product was sequenced on an ABI Prism 310 DNA automated sequencer, using the BigDye Terminator Cycle Sequencing Ready Reaction Kit version 3.1 (Applied Biosystems, Foster City, CA, USA). The DNA sequences reported in this work have been deposited in the GenBank database (Accession Nos. KJ543701, KJ543702).

## Results

### Analysis of the Iceman Non-human Reads

**Figure 1:**
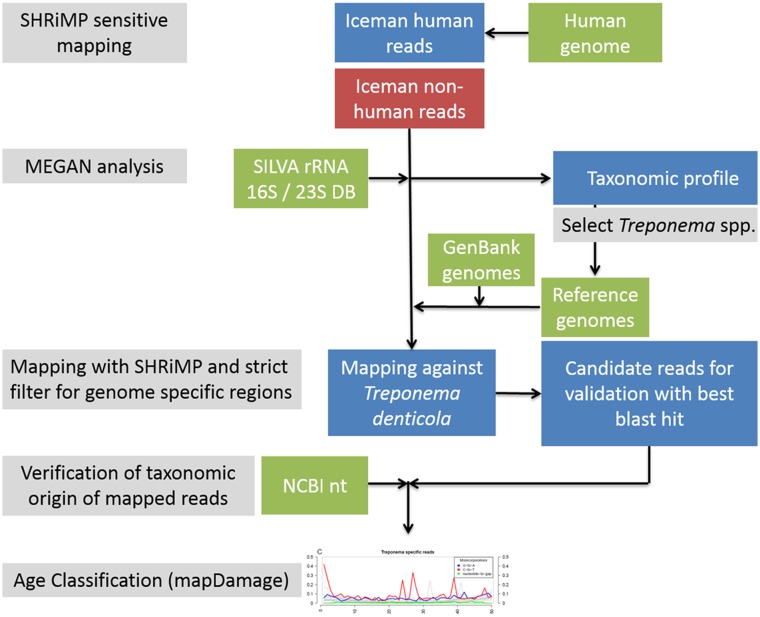
Schematic overview of the bioinformatics pipeline used in this study.

**Figure 2:**
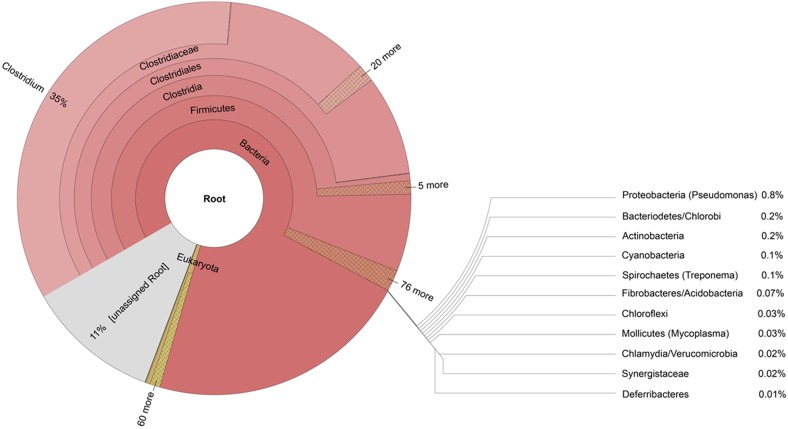
Taxonomic profile of the non-human Iceman metagenome. Phylogenetic assignment of the bacterial and eukaryotic rRNA reads of the Iceman’s metagenome to different phyla. Indicated in brackets are the predominant assignable genera within a phylum.

The Iceman genome study [15] created a massive metagenomic dataset, which consists of human and non-human reads. The metagenome was sequenced on a SOLID 4 sequencing platform producing short reads up to 50 bp with high accuracy. In this study we applied a bioinformatics pipeline on this SOLID sequencing data in order to accurately separate the human from the non-human reads and to further screen the non-human reads for potential ancient pathogens (Figure 1). To extract the non-human reads from the metagenome all reads were mapped against the human reference genome hg19 GRCh37 using the SHRiMP software package [36]. With parameters adapted to the unique nature of the Iceman metagenomic dataset (as outlined in the Appendix S1) out of the 1.1 billion total metagenomic reads, 0.9 billion could be mapped to the human reference genome. The remaining 0.2 billion non-human reads were further taxonomically classified using MEGAN [27] based on sequence-similarity searches against the small subunit (SSU) and large subunit (LSU) ribosomal RNA SILVA database. In total 371,905 reads of the 0.2 billion non-human reads were assigned to various bacterial and eukaryotic phyla down to the genus level (Figure 2). The bacterial fraction of reads, comprising 88% of all assigned reads, is highly dominated by the phylum *Firmicutes* (73% of the *Bacteria* reads) with the genus *Clostridium* being the major representative of this phylum (54% of the *Firmicutes* reads). The remaining 28% of the bacterial reads were mainly unassigned (25% of the *Bacteria* reads) or spread throughout numerous different bacterial phyla comprising 0.8% (*Proteobacteria*) or less of all assigned non-human reads. In the subsequent step, we focused on bacterial phyla with assigned reads at the genus level aiming to identify highly abundant genera which are dominated by potential human pathogens.

### Pre-selection of Bacterial Genera with Potential Human Pathogens

Based on the rRNA taxonomic profile (Figure 2) and following the pathogen classification of the NCBI microbial genome database we pre-selected assigned bacterial genera, which contain potential human pathogens and further ranked the genera by taxonomic abundance. The retrieved list was divided into a high abundance (more than 5000 assigned rRNA reads) and a low abundance group (less than 5000 assigned rRNA reads) of bacterial genera (Table 1). The listed genera containing human-pathogenic bacteria represent reasonable candidates for Iceman-associated potential pathogens and were then selected for further analysis on species level. Initially, the low abundance group was excluded from all further analysis since the low overall amount of assigned rRNA reads suggested only a minor presence of specific genomic reads which were not sufficient for a meaningful reconstruction of genomic sequences. Furthermore, the first two genera of the high abundance group, *Clostridium* and *Pseudomonas*, were dominated by environmental non-pathogenic bacterial species and thus these genera were not considered for further analysis.

**Table 1:** Assignment of bacterial rRNA reads of the Icemańs metagenome to different genera and pre-selection of human pathogenic or opportunistic pathogenic bacteria according to the NCBI Genome Project database (ftp://ftp.ncbi.nih.gov/genomes/genomeprj_archive/lproks_0.txt).

Genus	AssignedrRNA reads	Pathogenic speciesin the Icemannon-humanreads	
*Clostridium*	Genus: 32010, all*Clostridiales*: > 1Mio	Species mainlynon-pathogenic	**High abundance group**
*Pseudomonas*	8939	Mostly non-pathogenic(e.g. *P. flourescens*)	
*Treponema*	5503	Strong signal for theopportunistic oralpathogen *T. denticola*	
*Burkholderia*	1092	Plant pathogens	**High** **abundance group**
*Bacillus*	845	No humanpathogenic *Bacilli*	
*Mycoplasma*	389	Most to*M. arthritidis*	
*Erysipelothrix*	171	*Erysipelothrix* *rusiopathiae*	
*Fusobacterium*	78	*Fusobacterium*sp. 3_1_5R	
*Legionella*	70		
*Borrelia*	36		
*Helicobacter*	35		

The third most abundant genus with a total of 5,503 assigned rRNA reads is *Treponema*, which is known to contain different human pathogenic species [37]. Nearly all of the reads could be assigned to the human oral bacterium *T. denticola.* To further support this first rRNA-based indication for the presence of human opportunistic pathogen DNA in the metagenomic dataset we decided to focus in the following genome wide survey on the reads specific for the genus *Treponema*.

Genus specific regions of the currently available complete genomes of *Treponema* have been determined from genome alignments to all other published genome sequences from NCBI GenBank [38], retaining only regions having no similarities to any other genome outside the genus. Consequently, Iceman non-human reads mapped with SHRiMP to those of specific regions provided evidence for the presence of the respective genus in the metagenome. Interestingly, 15,670 reads were assigned to specific regions in the genomes from the genus *Treponema,* of which the majority of the reads (8,090) were mapped against the genome of the human opportunistic pathogen *T. denticola* (Figure S1).

### Analysis of the *Treponema denticola* Specific Reads

**Figure 3:**
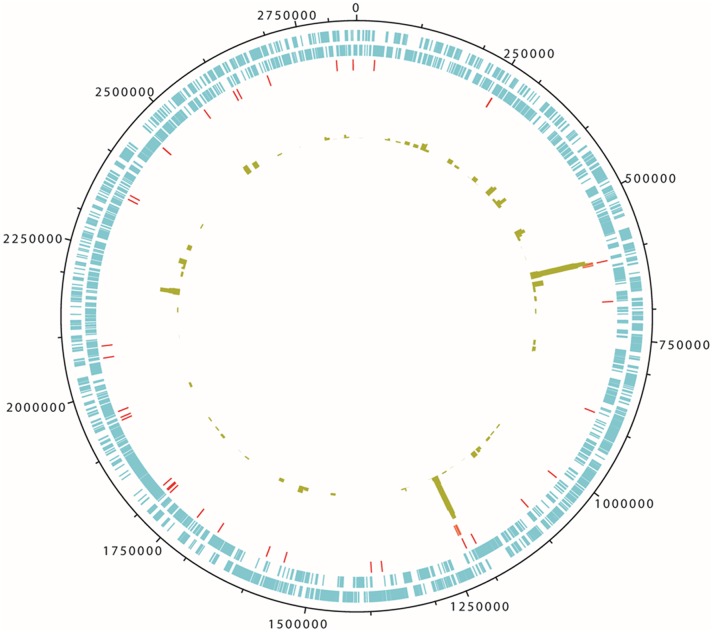
Gene coverage and distribution of the validated Iceman *Treponema denticola* reads mapped on the 2.8 Mb large genome of *T. denticola* ATCC35405. From outer to inner circles coding sequences forward and reverse are highlighted in blue, tRNA and rRNAs in red, and depicted by the green bars are the log scale coverage of mapped reads. For details on genes with mapped reads, please refer to Table S2.

For all further analyses the *T. denticola* specific reads were extracted from the Iceman non-human reads and were first mapped to the *T. denticola* ATCC 35405 genome (Figure 3). Even though the low number of reads excluded a reconstruction of the complete genome, the reads display an even distribution throughout the genome with an accumulation of reads in the two 23S rRNA gene copies (for details on the mapped gene loci please refer to the Table S2).

**Figure 4:**
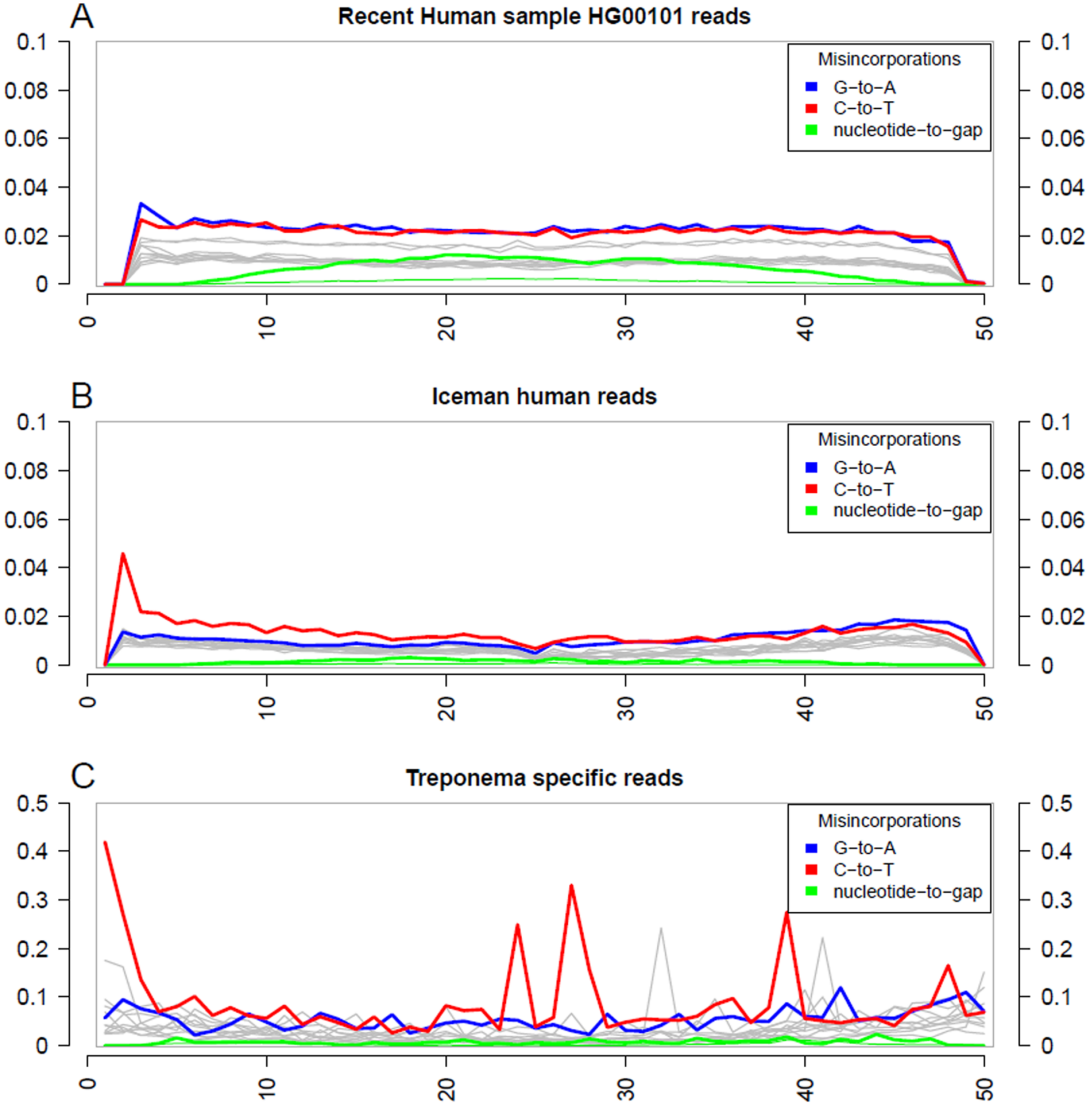
MapDamage analysis displaying the frequency of nucleotide misincorporations (y-axes) in SOLID reads (starting on the 5’-end, x-axes) of different datasets. (**A**) Human reference genome (ENA Experiment Accession No.: ERX008207) (**B**) Human reads of the Iceman metagenome (ENA Study Accession No.: ERP001144) (**C**) Validated *T. denticola* reads from the Iceman metagenome. Grey lines indicate all possible misincorporations; G-to-A and C-to-T misincorporations are plotted in blue and red, respectively. The green lines display all possible variants of a nucleotide-to-gap position.

To assess the nucleotide misincorporation patterns along the DNA fragments, a mapDamage analysis with the *T. denticola* specific reads was performed and compared to the damage pattern of the Iceman human reads (Figure 4). Furthermore, since the Iceman genome displays the first ancient genome sequenced on a SOLID platform [15], a modern human genome sequenced on the same platform was included as a control. Compared to the modern dataset the Iceman genome displays an increased C to T misincorporation pattern at the 5′ end of the reads (the absence of mismatches directly at the 5′ end of the human reads is a technical artefact resulting from the sensitive settings of the SHRiMP mapping tool). An increase of DNA damage was also observed in the *T. denticola* specific reads. However, the damage patterns occur in contrast to the Iceman genome at an order of magnitude higher frequency and the C to T misincorporation pattern is not restricted to the 5′ end and was found additionally within the reads.

**Figure 5:**
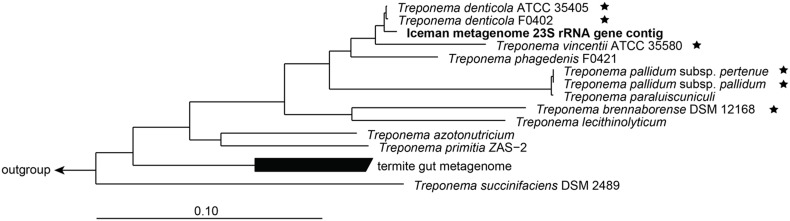
Phylogenetic tree based on bacterial 23S rRNA genes of the genus *Treponema* and selected bacteria of the phylum *Spirochaetes* (serving as an outgroup). The Iceman metagenome 23S rRNA contig is highlighted in bold. All sequences marked with an asterisk belong to a pathogenic or opportunistic pathogenic *Treponema* species. The scale bar indicates 10% estimated sequence divergence.

The 23S rRNA gene was the only phylogenetic marker gene sufficiently covered by *T. denticola* specific reads. Thus a contiguous consensus sequence of the 23S rRNA gene was extracted and used for further phylogenetic assignment. The partial Iceman *Treponema* 23S rRNA contig was first aligned against a subset of complete 23S rRNA sequences of the genus *Treponema*. In the next step, a phylogenetic tree based on 23S rRNA genes of bacteria of the genus *Treponema* and selected bacteria of the phylum *Spirochaetes*, which served as an outgroup, was calculated and the partial Iceman metagenome 23S rRNA gene contig was added to the tree using the Parsimony tool in the ARB software package [31]. In the resulting phylogenetic framework the partial Iceman metagenome 23S rRNA gene contig clustered together with sequences of *T. denticola* strains opening a new basal branch highly related to the *T. denticola* sequences (Figure 5).

### Molecular Screening for Opportunistic Pathogens in the Iceman’s Oral Cavity

**Figure 6:**
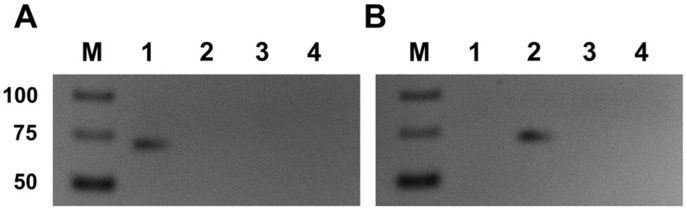
PCR-based detection of different opportunistic oral pathogens in Iceman’s gingival tissue (1) and mouth swab (2) samples. (**A**) PCR assay targeting the 16S rRNA gene of *T. denticola*. (**B**) PCR assay targeting the repetitive element IS1126 of *Porphyromonas gingivalis*. All assays include a PCR negative control (3) and a PCR of the DNA extraction blank (4).

The extraordinary well-preserved mummy provides the unique opportunity to confirm the presence of members of the human commensal oral microflora in the Iceman’s mouth region (Figure S2A). Therefore, the survey was extended to an Iceman gingival tissue sample and a mouth swab sample using a PCR-based diagnostics assay (Figure S2B). Both the 16S rRNA based PCR assay for *T. denticola* and the PCR assay targeting the repetitive element IS1127 of *Porphyrimonas gingivalis* gave a positive result. This indicates the presence of opportunistic pathogens in the Iceman gingival tissue and in the mouth swab sample, respectively (Figure 6). In this context, it is important to highlight that only the small IS1127 fragment of both applied IS1127 PCR assays was amplifiable which suggests the degraded nature of the *Porphyrimonas gingivalis* DNA (data not shown).

## Discussion

Here we report the discovery of sequence reads indicating the presence of opportunistic pathogens in Iceman’s tissue biopsies. In-depth analysis of the metagenome of the Iceman’s genomic survey [15] resulted in the recovery of *T. denticola* specific sequence reads which were further subjected to phylogenetic assignment and DNA damage pattern analysis. Finally, we extended our survey to an Iceman’s gingival tissue biopsy and mouth swab sample to detect, via conventional PCR, members of the human commensal oral microflora.

Initially we taxonomically classified the non-human reads of the Iceman’s metagenome by screening for rRNA specific reads. rRNA-based profiling has been increasingly applied to the taxonomic classification of bacteria in metagenomic studies of recent and ancient DNA datasets [23], [39]. Due to the specific sequence curation and alignment procedures used, SILVA-derived rRNA datasets provide fast computation and accurate taxon assignment [40].

Our taxonomic profiling revealed a high abundance of bacterial rRNA reads assigned to the genus *Clostridium* in the predominant phylum *Firmicutes* (Figure 2). The presence of Clostridia in Iceman’s tissue and intestinal samples was indicated in earlier molecular studies [41], [42] and our finding is consistent with previous data from the Iceman genomic survey [15]. Recently, metagenomic analysis discovered a high percentage of *Firmicutes* also in tissue samples of Egyptian mummies [43]. Moreover, a microbial survey of the mummies from the Capuchin Catacombs in Sicily, Italy, indicates that members of the order *Clostridiales* are predominant on mummified skin and muscle samples [44]. Presumably, these Clostridia-like bacteria display remnants of the post-mortem growth of the bacterial community, which is involved in the overall body decomposition process [45], [46]. With the present data, however, it is impossible to predict whether these spore-forming bacteria are still viable and could start to grow under favourable environmental conditions on the mummified tissues, thus increasing the risk for biodeterioration of these precious human remains.

Unexpectedly, after ranking the bacterial rRNA reads by abundance, we detected in addition to the numerous *Clostridium*-specific reads a high number of reads affiliated to the genus *Treponema* of which nearly all were assigned to the human oral spirochete *T. denticola. T. denticola* is an opportunistic pathogen, which is a member of the human commensal oral microflora. Whenever the oral microbiome is in a state of disequilibrium or when environmental conditions change within the host, *T. denticola* can promote a pathology as part of a microbial consortium. To further support the initial rRNA-based results, we extended our survey to whole genomes available for the genus *Treponema*. By mapping the non-human reads against regions having no similarities to any other genome outside the genus *Treponema* we obtained 8,090 reads mapping against the genome of the human opportunistic pathogen *T. denticola.* Due to the limited dataset we observed an uneven mapping of the reads to the genome with a high read abundance within the 23S rRNA gene. One possible explanation for the accumulation of reads in the conserved and highly variable regions of the ribosomal rRNA genes could be the presence of several *T. denticola* strains in the metagenome. However, we only see this read accumulation in the two 23S rRNA gene copies and not in the 16S rRNA genes, where we would have expected a similar but less pronounced effect, due to the shorter length of the 16S rRNA gene. Thus for phylogenetic assignment we decided to use a contiguous consensus sequence of the 23S rRNA gene to verify the presence of *Treponema* sequence reads in the reads mapped to the 23S rRNA gene. Furthermore, we performed a DNA damage pattern analysis on all obtained *Treponema* reads and compared the pattern to nucleotide misincorporation frequencies in the Iceman genomic reads. In contrast to the reads of the modern human reference genome both *Treponema* reads and Iceman genomic reads display increased frequency of C to T substitutions towards the fragment ends. It is important to note that the DNA library of the Iceman genomic survey [15], similar to the library of the Shaqqaq genome study [14], was PCR amplified with a Phusion Polymerase. This is a modified Pfu Polymerase [47], which has been demonstrated to show poor activity at uracil and/or deaminated cytosine residues [48], [49]. Therefore, the observed C to T misincorporation events in the Iceman genomic reads presumably indicate both conserved ancient methylation patterns as these have been recently reported in the Shaqqaq genomic reads [50], and diminished yet detectable levels of cytosine deamination [20]. Unexpectedly, the observed nucleotide misincorporation frequencies are much higher in the *T. denticola* reads than in the Iceman genomic reads. A similar quantitative effect has been reported in a recent ancient genome study on *Mycobacterium leprae*, the causative agent of leprosy [18]. In contrast to our observation in the *Treponema* study the DNA damage patterns in the leprosy study were much less pronounced in the bacterium than in the human host. The observed different nucleotide misincorporation frequencies require further investigation having more enriched *T. denticola* sequence data available. Nevertheless, the obtained *T. denticola* DNA sequences from our study indicates an already increased frequency of C to T substitutions close to the fragment ends characteristic of ancient DNA.

The detection of metagenomic reads specific for an opportunistic oral pathogen in the Iceman bone biopsy was unexpected and we provide below different explanatory models for this finding. One possible explanation for the presence of *T. denticola* reads in the bone biopsy could be the haematogenous spread of opportunistic oral pathogens as reported in recent literature [51], [52]. In addition, *T. denticola* and other members of the human commensal oral microflora seem to be associated with the formation of atherosclerotic plaques [53], [54]. Interestingly, the Iceman shows strong signs of generalized arteriosclerotic disease by the observation of several calcified plaques as previously revealed by CT scan analysis [55] (Figure S3). Since sampling of a plaque would be invasive, we decided to extend our survey to the mouth region of the Iceman, the actual living environment of opportunistic oral pathogens. Using a PCR-based assay, we detected the DNA of opportunistic oral pathogens in an Iceman’s gingival tissue and swab sample. Our results indicate the presence of bacteria of the oral microbiome in different Iceman tissue biopsies. The detection of opportunistic oral pathogens in the Iceman’s mouth region is in accordance with the findings of Adler and colleagues [56] and Warinner and colleagues [57], who could demonstrate the presence of oral microbiota DNA in numerous ancient calcified human dental plaques. Both *T. denticola* and *P. gingivalis* belong, together with *Tannerella forsythia*, to the so-called “red complex” bacteria which are members of the dental plaque biofilm community and which are highly associated with periodontal disease [58]–[60]. Periodontitis is the major cause of tooth loss worldwide. Bacterially induced chronic inflammatory processes can result in localized alveolar bone loss around the tooth root surfaces [58]. Recently a re-evaluation of CT scans of the Iceman focusing on the oral cavity could show extensive alveolar bone loss [61] indicative of periodontitis. Thus, our molecular data is in accordance with the CT-based results. Another possible explanation for the presence of *T. denticola* reads in the bone biopsy is beside the above mentioned haematogenous spread of bacteria during lifetime the dissemination of commensal microflora via the bloodstream around the body shortly before death. However, with the currently available data we cannot determine whether the spread of these opportunistic pathogens occurred during the Iceman’s lifetime or shortly before death.

Taken together, our data indicates the presence of bacteria of the oral microbiome in different Iceman tissue biopsies. Moreover, the obtained *T. denticola* DNA displays damage patterns characteristic for ancient DNA. The detection of the low-GC bacterium *T. denticola* may pave the way for future genome studies of important ancient outright pathogens within the genus *Treponema* such as *Treponema pallidum*, the causative agent of syphilis. Even more importantly, this study underscores the opportunity to detect disease-associated microorganisms when applying metagenomics-enabled approaches on datasets from ancient human remains.

## Supporting Information

Table S1 **Detailed list of all oligonucleotide primers used in this study and the corresponding PCR conditions.**
(DOCX)

Table S2 **Detailed list of the gene coverage of all validated** ***Treponema denticola*** **reads.**
(DOCX)

Figure S1 **Number of Iceman metagenomic reads specifically mapped to all available genomes of the genus** ***Treponema*** **.**
(TIF)

Figure S2 **(A) Iceman’s mouth region. Samples have been taken from the Iceman’s right oral cavity. (B) A gingival soft tissue sample (1) and a mouth swab sample (2) have been taken.**
(TIF)

Figure S3 **CT image of the Iceman’s abdomen. The arrows highlight two calcifications constituting aortic plaques in the aortic bifurcation.**
(TIF)

Appendix S1 **Supplementary Material and Methods,** ***In silico*** **analysis of the Iceman’s metagenome.**
(DOCX)
