# Supplementary material for: Metagenomic Analysis Reveals Presence of Treponema denticola in a Tissue Biopsy of the Iceman
Source: PLoS One. 2014 Jun 18;9(6):e99994. doi: 10.1371/journal.pone.0099994 (PMC4062476; doi:10.1371/journal.pone.0099994)
Supplement: Table S1 — Detailed list of all oligonucleotide primers used in this study and the corresponding PCR conditions. (DOCX) [file pone.0099994.s001.docx]

**SUPPLEMENTAL MATERIAL**

| **Moleculartarget** | **Primer** | **Sequence (5´-3´)** | **Product size (bp)** | **PCR conditions** | **Reference** |
| --- | --- | --- | --- | --- | --- |
| *Treponema denticola* 16S rRNA gene | Trepo16SF138 | TAATACCGAATGTGCTCATTTACAT | **68** | 95°C, 5 min;  95°C, 60°C, and 72°C, 45 sec  for 45cycles;  72°C, 4 min | [[1](#_ENREF_1)] and this study |
|  | Trepo16SR206 | CCCATCCTGAAGCGGAGCCGTAG |  |  |  |
| *Porphyrimonas gingivalis* IS1126 | PG279F | CACCTTTTCCCGATTCTTGGG | **71** | 95°C, 5 min;  95°C, 55°C, and 72°C, 1 min  for 50 cycles;  72°C, 4 min | This study |
|  | PG350R | GAAATCGACTGATGGTGCTGTGG |  |  |  |
| *Porphyrimonas gingivalis* IS1126 | PG244F | GATTGTGCTTTGGAGGAGCGC | **98** | 95°C, 5 min;  95°C, 55°C, and 72°C, 1 min  for 50 cycles;  72°C, 4 min | This study |
|  | PG342R | ACTGATGGTGCTGTGGTCGGG |  |  |  |

**Table S1:** Detailed list of all oligonucleotide primers used in this study and the corresponding PCR conditions.

1. Martinez-Pabon MC, Restrepo-Ospina DP, Isaza-Guzman DM, Orozco-Rojas LM, Tobon-Arroyave SI (2008) Detection of Treponema denticola in saliva obtained from patients with various periodontal conditions. Clin Oral Investig 12: 73-81.
