# Supplementary material for: Metagenomic Analysis Reveals Presence of Treponema denticola in a Tissue Biopsy of the Iceman
Source: PLoS One. 2014 Jun 18;9(6):e99994. doi: 10.1371/journal.pone.0099994 (PMC4062476; doi:10.1371/journal.pone.0099994)
Supplement: Table S2 — Detailed list of the gene coverage of all validated Treponema denticola reads. (DOCX) [file pone.0099994.s002.docx]

**SUPPLEMENTAL MATERIAL**

**Table S2:** Detailed list of the gene coverage of all validated *Treponema denticola* reads.

| **Description** | **begin** | **end** | **strand** | **locusTag** | **average_Coverage** | **#reads** |
| --- | --- | --- | --- | --- | --- | --- |
| 23S ribosomal RNA | 612128 | 615037 | + | TDE_23SA | 234.498 | 3850.46 |
| 23S ribosomal RNA | 1221739 | 1224648 | + | TDE_23SB | 104.332 | 1243.64 |
| ribonucleoside-diphosphate reductase%2C beta subunit | 374210 | 375265 | - | TDE_0335 | 548.547 | 822.82 |
| aminoacidpermease | 499884 | 501164 | + | TDE_0445 | 233.629 | 327.08 |
| hydro-lyase%2C tartrate%2Ffumarate family%2C betasubunit | 1567351 | 1567914 | + | TDE_1521 | 186.188 | 256.94 |
| radical SAM domainprotein | 2557502 | 2558329 | + | TDE_2530 | 168.676 | 249.64 |
| hydrogenaseaccessoryproteinHypB | 2197143 | 2197805 | - | TDE_2165 | 113.522 | 208.88 |
| oligopeptide%2Fdipeptide ABC transporter%2C ATP-bindingprotein | 1107076 | 1108116 | + | TDE_1076 | 126.205 | 184.26 |
| precorrin-8X methylmutase | 2581753 | 2582376 | + | TDE_2555 | 110.16 | 165.24 |
| conservedhypotheticalprotein | 635072 | 636100 | - | TDE_0596 | 47.174 | 135.86 |
| ABC transporter%2C ATP-bindingprotein | 410139 | 411695 | - | TDE_0364 | 82.148 | 133.08 |
| coenzyme A disulfide reductase%2C putative | 174578 | 176275 | - | TDE_0153 | 28.921 | 124.94 |
| transcriptional regulator%2C Sir2 family | 2312820 | 2313575 | + | TDE_2277 | 57.722 | 91.2 |
| methylaspartate mutase%2C E subunit | 2274750 | 2276207 | - | TDE_2236 | 26.44 | 70.86 |
| formiminotransferase%2C putative | 326932 | 327831 | - | TDE_0296 | 42.308 | 55 |
| pyridine nucleotide-disulphideoxidoreductase family protein | 155067 | 156437 | + | TDE_0135 | 39.75 | 50.88 |
| pyridine nucleotide-disulphideoxidoreductase family protein | 2198193 | 2200937 | - | TDE_2167 | 7.774 | 46.8 |
| conservedhypotheticalprotein | 415624 | 416643 | - | TDE_0367 | 34.6 | 44.98 |
| 16S ribosomal RNA | 1219839 | 1221354 | + | TDE_16SB | 5.086 | 44.96 |
| hypotheticalprotein | 1621003 | 1621572 | + | TDE_1572 | 28.667 | 37.84 |
| 16S ribosomal RNA | 610211 | 611726 | + | TDE_16SA | 5.479 | 37.26 |
| tRNA-Ser | 250688 | 250774 | - | TDE_tRNA-Ser-1 | 19.655 | 34.2 |
| ironcompound ABC transporter%2C ATP-binding protein%2C putative | 781021 | 782199 | - | TDE_0746 | 15.088 | 30.78 |
| Tplprotein | 132755 | 133669 | + | TDE_0113 | 18.113 | 25.72 |
| pyridoxinebiosynthesisprotein | 512626 | 513474 | - | TDE_0456 | 14.493 | 20 |
| conservedhypotheticalprotein | 400198 | 400833 | - | TDE_0357 | 14.406 | 19.88 |
| methylaspartate mutase%2C S subunit | 1055263 | 1055682 | + | TDE_1023 | 7.851 | 19 |
| precorrin-4 C11-methyltransferase | 648127 | 648876 | + | TDE_0614 | 14.754 | 18 |
| tRNA-Sec | 1090480 | 1090567 | + | TDE_tRNA-SeC(p)-1 | 13.857 | 17.46 |
| enoate reductase%2C putative | 2242665 | 2244845 | - | TDE_2205 | 5.152 | 17 |
| HAD-superfamily hydrolase%2C subfamily IA | 2773335 | 2773991 | + | TDE_2716 | 14.155 | 16.42 |
| desulfoferrodoxin%2Fneelaredoxin | 1806727 | 1807113 | - | TDE_1754 | 13.3 | 15.96 |
| glycinereductasecomplexproteinGrdD | 271918 | 273072 | - | TDE_0239 | 10.621 | 14.02 |
| glucose-1-phosphate thymidylyltransferase | 1485493 | 1486368 | + | TDE_1440 | 10.656 | 13 |
| tRNA-His | 1960359 | 1960430 | - | TDE_tRNA-His-1 | 8.778 | 12.64 |
| iron compound ABC transporter%2C periplasmic iron compound-binding protein%2C putative | 798018 | 799013 | - | TDE_0758 | 9.045 | 12.12 |
| TPR domainprotein | 502898 | 505876 | + | TDE_0447 | 9.677 | 12 |
| hypotheticalprotein | 1194507 | 1195931 | - | TDE_1160 | 5.14 | 11 |
| flagellar hook assembly scaffolding protein | 2822613 | 2823122 | - | TDE_0636 | 7.812 | 10 |
| oligopeptide%2Fdipeptide ABC transporter%2C ATP-bindingprotein | 670622 | 671590 | - | TDE_2769 | 5.682 | 10 |
| L-serine dehydratase%2C iron-sulfur-dependent%2C betasubunit | 1555152 | 1555814 | - | TDE_1507 | 6.359 | 9.92 |
|  | 250775 | 250821 |  | igr | 22.045 | 9.7 |
| KHG%2FKDPG family aldolase%2Fcarbohydrate kinase%2C PfkB family | 183999 | 185771 | + | TDE_0157 | 7.377 | 9 |
| oligopeptide%2Fdipeptide ABC transporter%2C ATP-bindingprotein | 1306080 | 1307066 | - | TDE_1272 | 5.933 | 8.9 |
| cysteinyl-tRNAsynthetase | 109867 | 111345 | - | TDE_0092 | 6.123 | 7.96 |
| leucyl-tRNAsynthetase | 2374985 | 2377561 | + | TDE_2339 | 3.465 | 7.9 |
|  | 2263859 | 2263877 |  | igr | 20 | 7.6 |
| tRNA-Leu | 1724782 | 1724864 | + | TDE_tRNA-Leu-2 | 6.649 | 7.58 |
| excinuclease ABC%2C A subunit | 1035367 | 1038231 | + | TDE_1010 | 6.364 | 7 |
| translationinitiationfactor IF-2 | 1138282 | 1140972 | - | TDE_1106 | 3.153 | 7 |
| oligopeptide%2Fdipeptide ABC transporter%2C ATP-bindingprotein | 1014181 | 1015137 | + | TDE_0987 | 6.123 | 6.98 |
| conservedhypotheticalprotein | 2263878 | 2264762 | + | TDE_2222 | 14.381 | 6.04 |
| conservedhypotheticalprotein | 2262824 | 2263858 | + | TDE_0665 | 11.577 | 6.02 |
| pyruvate ferredoxin%2Fflavodoxin oxidoreductase family protein | 699882 | 703442 | - | TDE_2221 | 2.408 | 6.02 |
| glycinereductasecomplexproprotein GrdE2 | 2152795 | 2154084 | - | TDE_0072 | 5 | 6 |
| hydrolase%2C carbon-nitrogen family | 1830047 | 1830934 | - | TDE_1793 | 3.896 | 6 |
| methyl-acceptingchemotaxisprotein | 81061 | 83199 | + | TDE_2120 | 2.752 | 6 |
| urocanatehydratase | 2646587 | 2648611 | + | TDE_2606 | 2.521 | 6 |
| choline%2Fcarnitine%2Fbetaine transporter family protein | 1295733 | 1297301 | + | TDE_1261 | 3.402 | 5.58 |
| translationelongationfactor G | 313762 | 315813 | + | TDE_0285 | 4.736 | 5.02 |
| CoA-substrate-specific enzyme activase domain protein | 1749588 | 1754090 | - | TDE_1695 | 2.282 | 5.02 |
| phage minor structural protein%2C putative | 1171558 | 1179627 | - | TDE_0078 | 4.808 | 5 |
| flagellarbiosynthesisproteinFliP | 2815156 | 2815971 | - | TDE_0581 | 4.545 | 5 |
| celldivisionproteinFtsZ | 1236443 | 1237726 | + | TDE_1142 | 4.386 | 5 |
| conservedhypotheticalprotein | 2558381 | 2558605 | + | TDE_1204 | 3.165 | 5 |
| transglutaminase-likedomainprotein | 616760 | 620476 | + | TDE_2531 | 2.315 | 5 |
| glycinereductasecomplexselenoprotein GrdB1 | 88835 | 90166 | - | TDE_2760 | 2.033 | 5 |
| carboxylesterasefamilyprotein | 566846 | 568342 | - | TDE_0521 | 4.623 | 4.9 |
| oligopeptide%2Fdipeptide ABC transporter%2C ATP-bindingprotein | 1095784 | 1096776 | - | TDE_1068 | 4.067 | 4.88 |
| hydrolase%2C carbon-nitrogen family | 1860033 | 1860920 | - | TDE_0012 | 3.39 | 4 |
| carbon starvationprotein CstA%2C putative | 16308 | 17762 | + | TDE_1827 | 3.333 | 4 |
| ribonucleoside-diphosphate reductase%2C alphasubunit | 379679 | 382216 | - | TDE_0341 | 3.518 | 3.94 |
| oligoendopeptidase F%2C putative | 2792903 | 2794633 | + | TDE_1364 | 3.518 | 3.94 |
| valyl-tRNAsynthetase | 1404903 | 1407632 | + | TDE_2738 | 1.99 | 3.94 |
| hypotheticalprotein | 338795 | 339700 | + | TDE_0307 | 3.217 | 3.86 |
| hypotheticalprotein | 2086399 | 2086599 | - | TDE_2059 | 4.735 | 3.22 |
| hypotheticalprotein | 185814 | 186038 | - | TDE_0158 | 2.576 | 3.04 |
| GMP synthase | 1772300 | 1773871 | + | TDE_0251 | 3 | 3 |
| ironcompound ABC transporter%2C ATP-binding protein%2C putative | 796188 | 796949 | - | TDE_0419 | 2.542 | 3 |
| hypotheticalprotein | 471063 | 472346 | + | TDE_0637 | 2.459 | 3 |
| transcriptionterminationfactorRho | 1551807 | 1553453 | + | TDE_0756 | 2.344 | 3 |
| tryptophanase | 284807 | 286186 | - | TDE_1503 | 1.5 | 3 |
| oligopeptide%2Fdipeptide ABC transporter%2C ATP-bindingprotein | 671583 | 672581 | - | TDE_1506 | 1.5 | 3 |
| L-serine dehydratase%2C iron-sulfur-dependent%2C alphasubunit | 1554242 | 1555132 | - | TDE_1718 | 1.456 | 3 |
| methyl-acceptingchemotaxisprotein | 539786 | 541906 | - | TDE_0484 | 1.38 | 2.98 |
| ABC transporter%2C ATP-binding%2Fpermease protein | 2832576 | 2834366 | - | TDE_1871 | 2.774 | 2.94 |
| membrane protein%2C putative | 1893752 | 1894612 | - | TDE_2782 | 1.455 | 2.94 |
| citrate lyase%2C beta subunit | 1681659 | 1682528 | - | TDE_1632 | 2.735 | 2.68 |
| tRNA-Met | 1395627 | 1395699 | - | TDE_tRNA-Met-2 | 3 | 2.28 |
| chaperonin%2C 33 kDafamily | 2580885 | 2581763 | + | TDE_2554 | 11.3 | 2.26 |
| conservedhypotheticalprotein | 21998 | 23026 | - | TDE_0017 | 1 | 2.02 |
| %28R%29-2-hydroxyglutaryl-CoA dehydratase%2C beta subunit%2C putative | 441936 | 443114 | - | TDE_0392 | 2 | 2 |
| hybrid clusterprotein | 535119 | 536750 | - | TDE_0479 | 2 | 2 |
| phosphonate ABC transporter%2C ATP-binding protein%2C putative | 950960 | 951730 | - | TDE_0928 | 2 | 2 |
| tRNA-Phe | 1789641 | 1789713 | + | TDE_1002 | 2 | 2 |
| tRNA-Arg | 1960253 | 1960326 | - | TDE_1194 | 2 | 2 |
| membrane protein%2C putative | 1028535 | 1029227 | - | TDE_1422 | 1.852 | 2 |
| CDP-alcohol phosphatidyltransferase family protein | 2706251 | 2707033 | - | TDE_tRNA-Phe-1 | 1.852 | 2 |
| galactoside ABC transporter%2C ATP-bindingprotein | 2255250 | 2256743 | - | TDE_1916 | 1.818 | 2 |
| glycosyl transferase%2C group 2 family protein | 1465885 | 1466910 | + | TDE_tRNA-Arg-3 | 1.786 | 2 |
| isoleucyl-tRNAsynthetase | 2698706 | 2702008 | - | TDE_2216 | 1.754 | 2 |
| glycerolkinase | 1933367 | 1934857 | - | TDE_2264 | 1.724 | 2 |
| ABC transporter%2C ATP-binding%2Fpermease protein | 2300333 | 2302123 | - | TDE_2421 | 1.449 | 2 |
| hypotheticalprotein | 1225113 | 1227683 | + | TDE_2663 | 1.099 | 2 |
| DNA-directed RNA polymerase%2C betasubunit | 2449349 | 2452852 | - | TDE_2669 | 1 | 2 |
| ATP-dependent DNA helicase%2C RecQ family | 489648 | 491351 | - | TDE_0435 | 1.65 | 1.98 |
| oligopeptide%2Fdipeptide ABC transporter%2C ATP-bindingprotein | 1013201 | 1014181 | + | TDE_0071 | 1.96 | 1.96 |
| peptidase%2C U32 family | 78742 | 80982 | + | TDE_0986 | 1.75 | 1.96 |
| BNR domainprotein | 2761784 | 2766448 | - | TDE_2709 | 1.42 | 1.96 |
| polyribonucleotidenucleotidyltransferase | 1068464 | 1070560 | + | TDE_1041 | 1 | 1.94 |
|  | 2086600 | 2086660 |  | igr | 4.13 | 1.9 |
|  | 610120 | 610210 |  | igr | 2.686 | 1.88 |
| methyl-acceptingchemotaxisprotein | 1080980 | 1083058 | + | TDE_1054 | 1.808 | 1.88 |
| hypotheticalprotein | 1858651 | 1859871 | - | TDE_1826 | 1.917 | 1.84 |
|  | 2772997 | 2773334 |  | igr | 7.667 | 1.38 |
| hypotheticalprotein | 1828398 | 1828865 | - | TDE_1791 | 1 | 1.1 |
|  | 1068349 | 1068463 |  | igr | 1 | 1.08 |
| flagellarbiosynthesisproteinFlhA | 63705 | 65801 | + | TDE_0055 | 1 | 1.06 |
| hypotheticalprotein | 439313 | 440110 | + | TDE_0388 | 1 | 1.04 |
| GGDEF domainprotein | 1737442 | 1740405 | - | TDE_1685 | 1 | 1.04 |
| exodeoxyribonuclease VII%2C large subunit | 1048075 | 1049271 | + | TDE_1017 | 1 | 1.02 |
| oligopeptide%2Fdipeptide ABC transporter%2C permeaseprotein | 1096773 | 1097789 | - | TDE_1069 | 1 | 1.02 |
| NQR2%2FRnfD%2FRnfE family protein | 1431115 | 1432149 | + | TDE_1391 | 1 | 1.02 |
| hypotheticalprotein | 1822273 | 1822398 | - | TDE_1780 | 1 | 1.02 |
| copper-translocating P-type ATPase | 11031 | 13706 | + | TDE_0008 | 1 | 1 |
| hypotheticalprotein | 128539 | 128847 | + | TDE_0108 | 1 | 1 |
| carbohydrate kinase%2C FGGY family | 156780 | 158339 | + | TDE_0137 | 1 | 1 |
| DNA-bindingresponseregulator | 169961 | 170566 | + | TDE_0149 | 1 | 1 |
| membrane protein%2C putative | 209881 | 210555 | - | TDE_0177 | 1 | 1 |
| iron-sulfur cluster-binding protein | 293874 | 294785 | + | TDE_0260 | 1 | 1 |
| membrane protein%2C putative | 355840 | 357285 | - | TDE_0323 | 1 | 1 |
| transporter%2C putative | 406129 | 407502 | + | TDE_0361 | 1 | 1 |
| ABC transporter%2C ATP-binding%2Fpermease protein | 426241 | 427812 | - | TDE_0373 | 1 | 1 |
| oligopeptide%2Fdipeptide ABC transporter%2C permeaseprotein | 444121 | 445083 | + | TDE_0394 | 1 | 1 |
| hypotheticalprotein | 472358 | 473545 | + | TDE_0420 | 1 | 1 |
| hypotheticalprotein | 566164 | 566751 | - | TDE_0520 | 1 | 1 |
|  | 571224 | 571580 |  | igr | 1 | 1 |
| methyl-acceptingchemotaxisprotein | 674621 | 676855 | - | TDE_0640 | 1 | 1 |
| sensorhistidine kinase%2C putative | 691737 | 692810 | + | TDE_0656 | 1 | 1 |
| conserveddomainprotein | 711250 | 712071 | + | TDE_0672 | 1 | 1 |
| hypotheticalprotein | 769286 | 770677 | - | TDE_0730 | 1 | 1 |
| translationelongationfactor Tu | 804872 | 806059 | + | TDE_0765 | 1 | 1 |
|  | 845967 | 846101 |  | igr | 1 | 1 |
| phosphohexosemutasefamilyprotein | 885384 | 887237 | + | TDE_0864 | 1 | 1 |
| membrane protein%2C putative | 887754 | 888689 | + | TDE_0866 | 1 | 1 |
| acetatekinase | 958203 | 959396 | + | TDE_0933 | 1 | 1 |
| hypotheticalprotein | 999061 | 999939 | + | TDE_0975 | 1 | 1 |
| tex protein%2C putative | 1041533 | 1043986 | + | TDE_1012 | 1 | 1 |
| hypotheticalprotein | 1263569 | 1265293 | - | TDE_1231 | 1 | 1 |
| batB protein%2C putative | 1285588 | 1286556 | + | TDE_1251 | 1 | 1 |
| oligopeptide%2Fdipeptide ABC transporter%2C permeaseprotein | 1308733 | 1309650 | - | TDE_1274 | 1 | 1 |
| signalpeptidase I | 1350790 | 1351326 | + | TDE_1313 | 1 | 1 |
| conservedhypotheticalprotein | 1372443 | 1373840 | + | TDE_1336 | 1 | 1 |
| conservedhypotheticalprotein | 1378582 | 1380621 | + | TDE_1342 | 1 | 1 |
| RNA polymerase sigma-70 factor family protein | 1385359 | 1387218 | + | TDE_1346 | 1 | 1 |
| methyl-acceptingchemotaxisprotein | 1425081 | 1426916 | + | TDE_1386 | 1 | 1 |
| aminotransferase%2C DegT%2FDnrJ%2FEryC1%2FStrS family | 1468759 | 1469865 | + | TDE_1426 | 1 | 1 |
| MutS2 familyprotein | 1495460 | 1497916 | + | TDE_1450 | 1 | 1 |
| conservedhypotheticalprotein | 1546853 | 1548160 | - | TDE_1500 | 1 | 1 |
| hypotheticalprotein | 1610863 | 1611471 | + | TDE_1559 | 1 | 1 |
| DNA polymerase I | 1686212 | 1689022 | + | TDE_1637 | 1 | 1 |
| dephospho-CoA kinase%2C putative | 1689022 | 1689693 | + | TDE_1638 | 1 | 1 |
| hypotheticalprotein | 1696880 | 1697527 | - | TDE_1649 | 1 | 1 |
| tRNA-Gly | 1724894 | 1724965 | + | TDE_tRNA-Gly-1 | 1 | 1 |
| membrane protein%2C putative | 1744181 | 1745881 | - | TDE_1688 | 1 | 1 |
| hypotheticalprotein | 1760728 | 1761264 | + | TDE_1704 | 1 | 1 |
| apolipoprotein N-acyltransferase | 1763278 | 1764912 | - | TDE_1708 | 1 | 1 |
| preprotein translocase%2C SecA subunit | 1918212 | 1920980 | - | TDE_1898 | 1 | 1 |
| 1-deoxy-D-xylulose-5-phosphate synthase | 1926966 | 1928927 | - | TDE_1910 | 1 | 1 |
| sigma-54 dependent transcriptional regulator%2Fresponse regulator | 1983169 | 1984590 | - | TDE_1969 | 1 | 1 |
|  | 2037623 | 2037913 |  | igr | 1 | 1 |
| aminoacid ABC transporter%2C aminoacid-binding protein%2C putative | 2118853 | 2119686 | + | TDE_2091 | 1 | 1 |
| ATP-dependent RNA helicase%2C DEAD%2FDEAH box family | 2133665 | 2135377 | - | TDE_2109 | 1 | 1 |
| conservedhypotheticalprotein | 2154202 | 2154819 | - | TDE_2121 | 1 | 1 |
| methyl-acceptingchemotaxisprotein | 2175909 | 2178047 | + | TDE_2142 | 1 | 1 |
|  | 2244846 | 2245010 |  | igr | 1 | 1 |
| CarBfamilyprotein | 2247729 | 2249360 | + | TDE_2209 | 1 | 1 |
| methylaspartateammonia-lyase | 2273440 | 2274678 | - | TDE_2235 | 1 | 1 |
| LysMdomainprotein | 2317510 | 2318583 | - | TDE_2281 | 1 | 1 |
| ribosomalprotein S2 | 2383593 | 2384468 | - | TDE_2347 | 1 | 1 |
| high-affinity branched-chain amino acid ABC transporter%2C ATP-binding protein | 2398269 | 2399039 | - | TDE_2364 | 1 | 1 |
| iron-sulfur cluster-binding protein | 2403723 | 2404556 | + | TDE_2371 | 1 | 1 |
| conservedhypotheticalprotein | 2426646 | 2427782 | + | TDE_2401 | 1 | 1 |
| shikimatekinase | 2476410 | 2477018 | - | TDE_2447 | 1 | 1 |
| conservedhypotheticalprotein | 2481577 | 2482161 | + | TDE_2453 | 1 | 1 |
| ABC transporter%2C ATP-binding%2Fpermease protein | 2534322 | 2536055 | + | TDE_2510 | 1 | 1 |
| lipoprotein%2C putative | 2565285 | 2568071 | - | TDE_2540 | 1 | 1 |
| methyl-acceptingchemotaxisprotein | 2573425 | 2575524 | + | TDE_2549 | 1 | 1 |
| hypotheticalprotein | 2644314 | 2645864 | - | TDE_2604 | 1 | 1 |
| conservedhypotheticalprotein | 2652120 | 2654339 | + | TDE_2611 | 1 | 1 |
|  | 2676094 | 2676209 |  | igr | 1 | 1 |
| methyl-acceptingchemotaxisprotein | 2834550 | 2836667 | + | TDE_2783 | 1 | 1 |
| conservedhypotheticalprotein | 2836978 | 2838687 | + | TDE_2784 | 1 | 1 |
| alcohol dehydrogenase%2C iron-containing | 60163 | 61314 | - | TDE_0051 | 1 | 0.98 |
| membrane protein%2C putative | 208890 | 209888 | - | TDE_0176 | 1 | 0.98 |
| conservedhypotheticalprotein | 247348 | 248325 | - | TDE_0212 | 1 | 0.98 |
| hypotheticalprotein | 373386 | 374138 | - | TDE_0334 | 1 | 0.98 |
| acetyl-CoA carboxylase%2C carboxyl transferase%2C betasubunit | 629155 | 629946 | - | TDE_0590 | 1 | 0.98 |
| oligopeptide%2Fdipeptide ABC transporter%2C ATP-bindingprotein | 1094634 | 1095791 | - | TDE_1067 | 1 | 0.98 |
| hypotheticalprotein | 1199039 | 1199572 | - | TDE_1168 | 1 | 0.98 |
| zinc ABC transporter%2C permeaseprotein | 1257930 | 1258844 | - | TDE_1224 | 1 | 0.98 |
| conserveddomainprotein | 1598927 | 1600129 | + | TDE_1556 | 1 | 0.98 |
| ATP-dependent Clp protease%2C ATP-binding subunit ClpX | 1727018 | 1728265 | + | TDE_1673 | 1 | 0.98 |
|  | 2269514 | 2269777 |  | igr | 1 | 0.98 |
| membrane protein%2C putative | 1072215 | 1073288 | + | TDE_1044 | 1 | 0.96 |
| conservedhypotheticalprotein | 1627579 | 1629816 | - | TDE_1580 | 1 | 0.96 |
| AMP-binding enzyme family protein | 1795743 | 1797668 | - | TDE_1742 | 1 | 0.96 |
| conservedhypotheticalprotein | 1981697 | 1982593 | + | TDE_1967 | 1 | 0.96 |
| responseregulator | 2524913 | 2525887 | + | TDE_2501 | 1 | 0.96 |
| hypotheticalprotein | 935417 | 939043 | + | TDE_0918 | 1 | 0.94 |
| hypotheticalprotein | 1576091 | 1576669 | + | TDE_1531 | 1 | 0.94 |
| UDP-N-acetylglucosamine--N-acetylmuramyl-%28pentapeptide%29 pyrophosphoryl-undecaprenol N-acetylglucosaminetransferase | 1987825 | 1989000 | - | TDE_1974 | 1 | 0.94 |
|  | 1100679 | 1100880 |  | igr | 1 | 0.92 |
| HAM1 protein | 298653 | 299198 | - | TDE_0267 | 1 | 0.88 |
|  | 753559 | 753601 |  | igr | 1 | 0.86 |
| hypotheticalprotein | 578154 | 578954 | - | TDE_0536 | 1 | 0.84 |
| triggerfactor | 1725052 | 1726407 | + | TDE_1671 | 1 | 0.82 |
|  | 1133769 | 1133825 |  | igr | 1 | 0.8 |
| phosphatase%2Fnucleotidase | 892838 | 894736 | + | TDE_0870 | 1 | 0.78 |
|  | 1656949 | 1657096 |  | igr | 1 | 0.76 |
|  | 2542254 | 2542543 |  | igr | 1 | 0.76 |
|  | 1395700 | 1395763 |  | igr | 3 | 0.72 |
|  | 685872 | 688316 |  | igr | 1 | 0.7 |
| glycogensynthase | 1630374 | 1631849 | - | TDE_1582 | 1 | 0.62 |
| hypotheticalprotein | 1886819 | 1887046 | - | TDE_1861 | 1 | 0.58 |
|  | 751805 | 751918 |  | igr | 1 | 0.56 |
|  | 611727 | 611868 |  | igr | 1 | 0.54 |
| aminoacyl-histidine dipeptidase%2C putative | 2267377 | 2268822 | - | TDE_2228 | 1 | 0.5 |
| membrane protein%2C putative | 952933 | 953778 | - | TDE_0930 | 1 | 0.48 |
|  | 953779 | 953850 |  | igr | 1 | 0.48 |
| CAAX amino terminal proteasefamilyprotein | 750968 | 751804 | + | TDE_0716 | 1 | 0.44 |
|  | 1724552 | 1724781 |  | igr | 3 | 0.42 |
| conservedhypotheticalprotein | 1886292 | 1886822 | + | TDE_1860 | 1 | 0.42 |
|  | 1631850 | 1631947 |  | igr | 1 | 0.4 |
|  | 2267195 | 2267376 |  | igr | 1 | 0.4 |
| citratelyaseacylcarrierprotein | 1682518 | 1682781 | - | TDE_1633 | 2 | 0.32 |
| membrane protein%2C putative | 684837 | 685871 | + | TDE_0650 | 1 | 0.3 |
| MutT%2Fnudix familyprotein | 1657097 | 1657621 | - | TDE_1607 | 1 | 0.24 |
| hypotheticalprotein | 2542161 | 2542253 | + | TDE_2516 | 1 | 0.24 |
|  | 892723 | 892837 |  | igr | 1 | 0.22 |
| hypotheticalprotein | 1132860 | 1133768 | - | TDE_1098 | 1 | 0.2 |
|  | 1724966 | 1725051 |  | igr | 1 | 0.18 |
|  | 1960431 | 1960573 |  | igr | 3 | 0.12 |
|  | 174568 | 174577 |  | igr | 1 | 0.12 |
| bacteriocin ABC transporter%2C ATP-binding%2Fpermease protein%2C putative | 753602 | 755755 | + | TDE_0719 | 1 | 0.1 |
|  | 1859872 | 1860032 |  | igr | 1 | 0.1 |
|  | 1090448 | 1090479 |  | igr | 1.333 | 0.08 |
|  | 298649 | 298652 |  | igr | 1 | 0.08 |
|  | 2557329 | 2557501 |  | igr | 1.5 | 0.06 |
|  | 1960327 | 1960358 |  | igr | 1 | 0.06 |
| conservedhypotheticalprotein | 297857 | 298648 | - | TDE_0266 | 1 | 0.04 |
|  | 1555815 | 1556115 |  | igr | 1 | 0.04 |
|  | 2524597 | 2524912 |  | igr | 1 | 0.04 |
|  | 248326 | 248407 |  | igr | 1 | 0.02 |
|  | 2558330 | 2558380 |  | igr | 1 | 0.02 |
