# Supplementary figures and images for: Metagenomic Analysis Reveals Presence of Treponema denticola in a Tissue Biopsy of the Iceman
Source: PLoS One. 2014 Jun 18;9(6):e99994. doi: 10.1371/journal.pone.0099994 (PMC4062476; doi:10.1371/journal.pone.0099994)

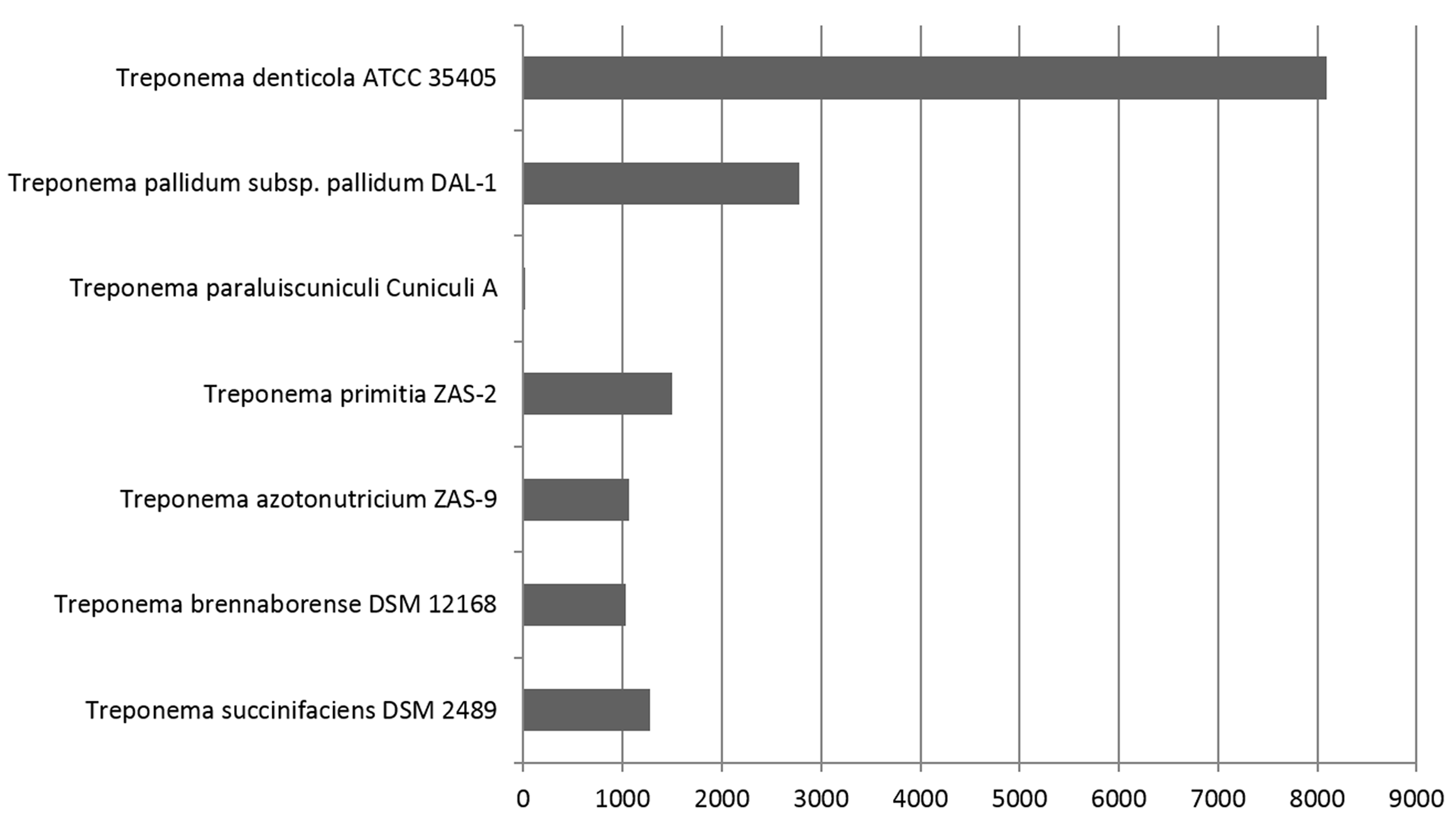

Supplement: Figure S1 — Number of Iceman metagenomic reads specifically mapped to all available genomes of the genus Treponema . (TIF) [file pone.0099994.s003.tif]

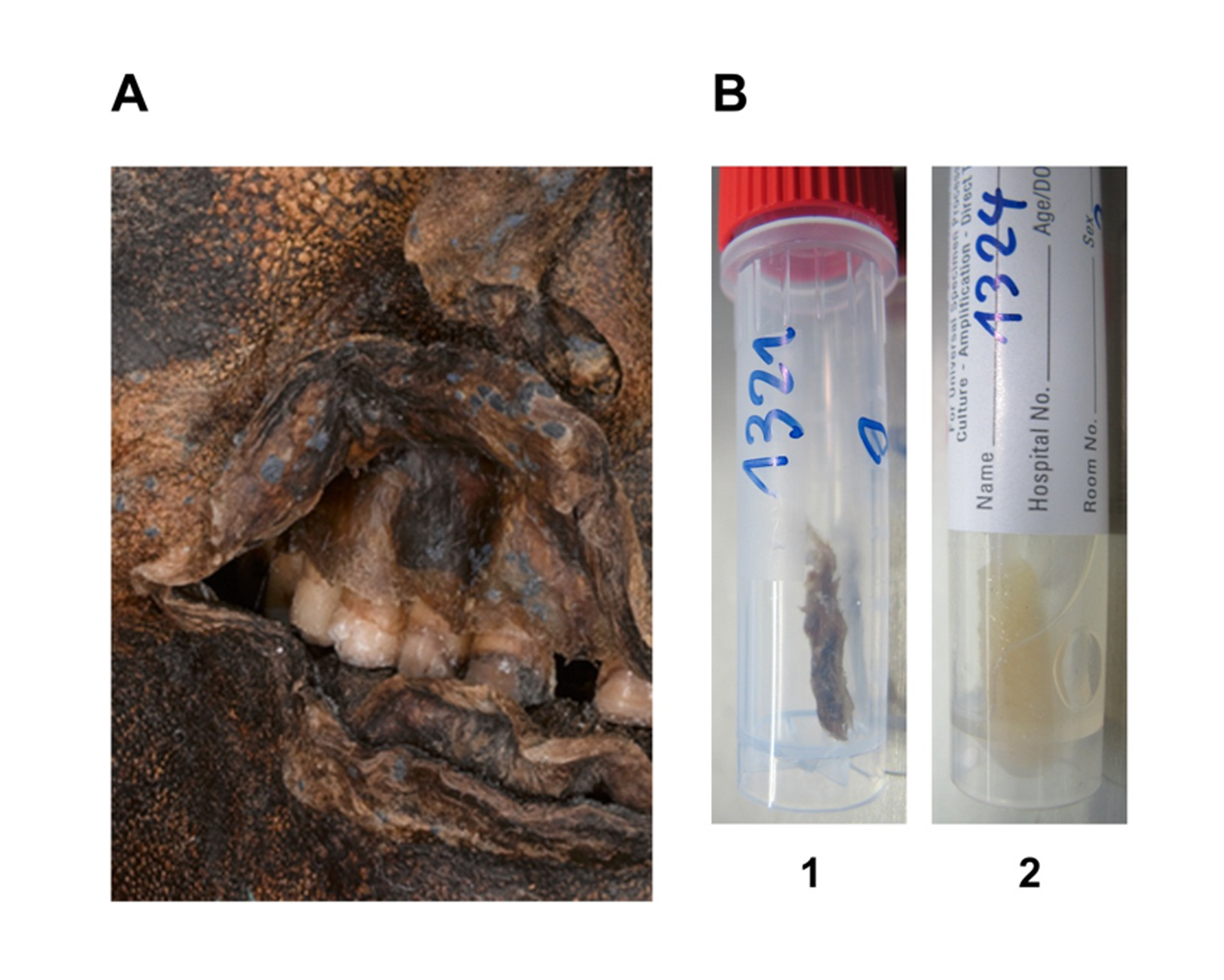

Supplement: Figure S2 — (A) Iceman’s mouth region. Samples have been taken from the Iceman’s right oral cavity. (B) A gingival soft tissue sample (1) and a mouth swab sample (2) have been taken. (TIF) [file pone.0099994.s004.tif]

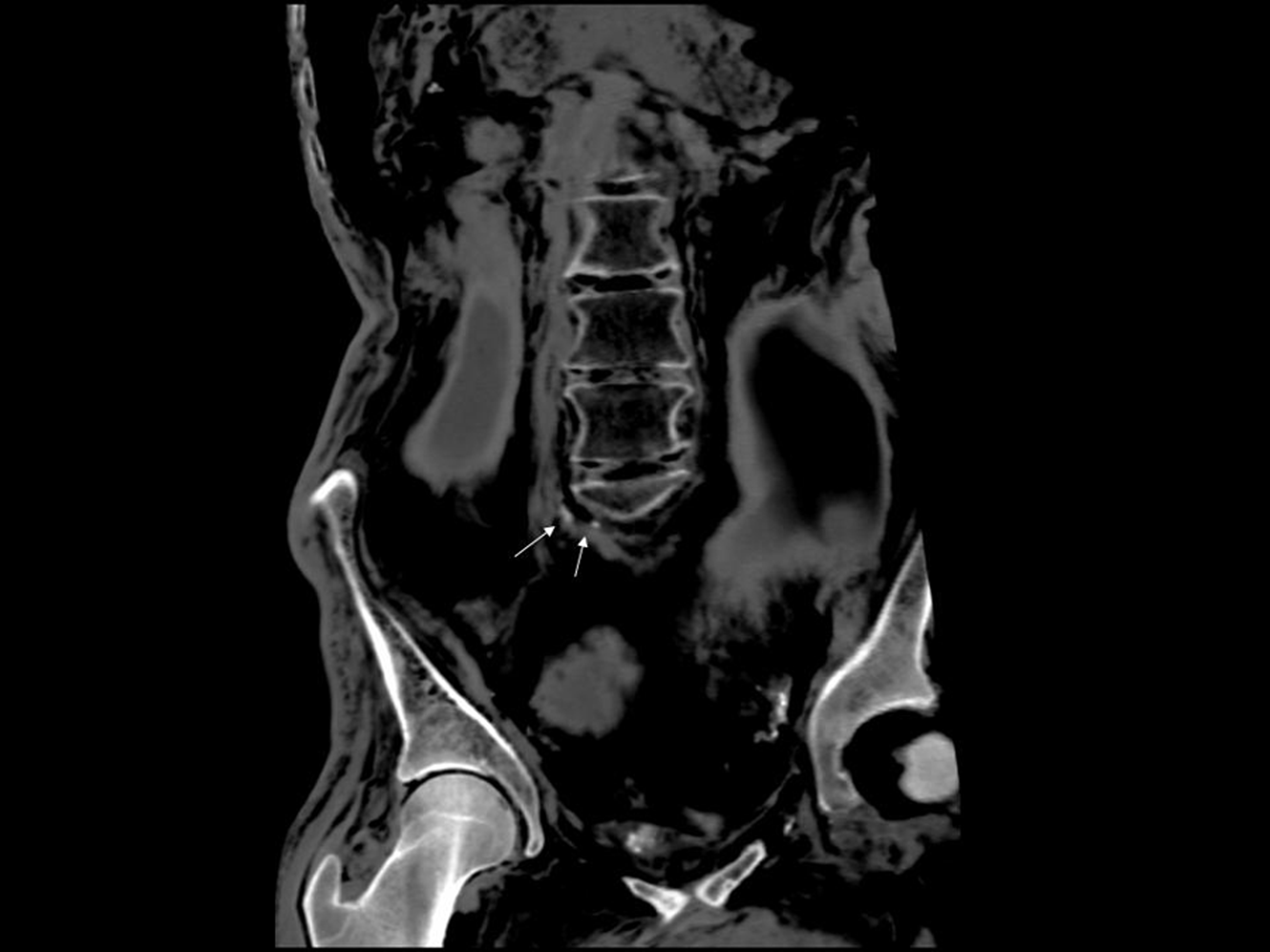

Supplement: Figure S3 — CT image of the Iceman’s abdomen. The arrows highlight two calcifications constituting aortic plaques in the aortic bifurcation. (TIF) [file pone.0099994.s005.tif]
