# Supplementary material for: Metagenomic Analysis Reveals Presence of Treponema denticola in a Tissue Biopsy of the Iceman
Source: PLoS One. 2014 Jun 18;9(6):e99994. doi: 10.1371/journal.pone.0099994 (PMC4062476; doi:10.1371/journal.pone.0099994)
Supplement: Appendix S1 — Supplementary Material and Methods, In silico analysis of the Iceman’s metagenome. (DOCX) [file pone.0099994.s006.docx]

**SUPPLEMENTAL MATERIAL**

**Appendix S1: Supplementary Material and Methods**

***In silico* analysis of the Iceman´s metagenome**

*Identification of the non-human reads in the Iceman´s metagenome*

The usage of SHRiMP [1] as mapping tool was evaluated for sensitivity. In previous unpublished work of a masters thesis, the evaluation of several established mapping tools focused on the sensitive detection of ancient Iceman reads in the mapping procedure. With the degradation pattern of ancient DNA remains and the short 50 nucleotide SOLiD reads an adapted procedure of the standard mapping process was established. The sensitivity was evaluated with a control in-silico experiment. The mismatch score for the mapping was iterated of each mapping comparing human reference genome and a negative control reference genome: *Nematostella vectensis* [2], which represents an animal species of which no close relatives are expected in the Iceman sample. For these alignments the e-value inferred from a BLAST alignment was used to establish a mismatch score threshold for significant alignments.

*Identification of Treponema specific reads*

To further evaluate the specificity of the assignment of the filtered reads against the Genus *Treponema* two databases were generated: one only containing *Treponema* specific sequences (nt_trep) and one "nt" nucleotide database without *Treponema* sequences (nt_notr). The nucleotide database (NT) from NCBI as a flat file of the fasta format was therefore filtered with an regular expression "^treponema .*" for the *Treponema* species. Taxonomically unclassified sequences were removed from both databases by matching against the regular expression ".*unclassified.*|.*uncultured.*|.*uncultured.*".

The reads, which mapped against the *Treponema* reference genome, were searched in both databases with limitation to the best hit (BLAST parameters: -b 1 -v 1 -e 0.1 -F F -m 8, returning only one hit with an e-value better than p=0.1 and using no complexity filter). For each read having a BLAST hit in the database nt_trep the score is compared to the best hit score in the nt_notr database. If the *Treponema* read hit is having a score higher than the same read in the other database nt_notr with a 10 percent margin [0.9*score(nt_trep) => score(nt_notr)], the read is considered specific for the genus *Treponema*.

The *Treponema*-specific reads were extracted from the mapping with a custom perl script. The generated “sam” file is used in the following steps.

**References**

1. Hormozdiari F, Hach F, Sahinalp SC, Eichler EE, Alkan C (2011) Sensitive and fast mapping of di-base encoded reads. Bioinformatics 27: 1915-1921.

2. Putnam NH, Srivastava M, Hellsten U, Dirks B, Chapman J, et al. (2007) Sea anemone genome reveals ancestral eumetazoan gene repertoire and genomic organization. Science 317: 86-94.
